# Nematodes and Microorganisms Interactively Stimulate Soil Organic Carbon Turnover in the Macroaggregates

**DOI:** 10.3389/fmicb.2018.02803

**Published:** 2018-11-26

**Authors:** Yuji Jiang, Hu Zhou, Lijun Chen, Ye Yuan, Huan Fang, Lu Luan, Yan Chen, Xiaoyue Wang, Manqiang Liu, Huixin Li, Xinhua Peng, Bo Sun

**Affiliations:** ^1^State Key Laboratory of Soil and Sustainable Agriculture, Institute of Soil Science, Chinese Academy of Sciences, Nanjing, China; ^2^University of Chinese Academy of Sciences, Beijing, China; ^3^Mudanjang Tobacco Science Research Institute, Harbin, China; ^4^College of Resources and Environmental Sciences, Nanjing Agricultural University, Nanjing, China

**Keywords:** nematode assemblages, microbial community, soil porosity, nutrient availability, carbon metabolic activities, soil organic carbon turnover, soil macroaggregates

## Abstract

The intra-aggregate architecture of soil macroaggregates provides suitable microhabitats for nematodes to graze on microorganisms. However, it is not fully clear how nematodes and microbial communities interactively mediate soil organic carbon (SOC) turnover. Here, we aimed to illustrate the relationships between nematodes, microbial community, and SOC turnover in the macroaggregates of a red soil receiving long-term manure application. Soil macroaggregates (>2 mm) were sampled from an 11-year field experiment including four manure treatments: no manure (M0), low manure rate (M1), high manure rate (M2), and high manure rate with lime (M3). The abundances of nematodes and microbial communities were substantially increased under manure treatments. Bacterivores dominated under the M2 and M3 treatments, while plant parasites were enriched under the M1 treatment. Phospholipid fatty acid analysis indicated that the ratio of bacteria to fungi significantly increased, but the ratio of Gram-positive bacteria to Gram-negative bacteria declined with the increasing manure addition. Random forest modeling showed that soil porosity had a primary effect on nematode assemblages, while pH and SOC contributed profoundly to the structure of the microbial community and carbon metabolic capacity. Structural equation modeling suggested that nematode grazing promoted carbon metabolic activities predominantly due to increased microbial biomass. Taken together, the mechanistic understanding of nematode-microorganism interactions may have important implications for improving soil fertility by nematode-mediated microbial processes.

## Introduction

Soil aggregation plays a critical role in soil organic carbon (SOC) sequestration in agricultural ecosystems (Lehmann and Kleber, 2015). Optimized aggregation is essential for the management of soil structure, as aggregation provides physical protection for organic matter and forms suitable microhabitats for microfauna and microorganisms. The sizes of macroaggregates depend on the intimate association between mineral particles and soil organic matter (Tisdall and Oades, 1982).

Manure-based soil amendments can greatly increase soil aggregation and simultaneously improve SOC sequestration and turnover via the accumulation of macroaggregate-protected carbon (Mikha and Rice, 2004). Soil macroaggregate formation substantially influences both the complexity of the spatial arrangement of a pore matrix and the distribution patterns of organic matter at microscales. Manure application can enhance the complexity of a pore network system and the connected porosity of macroaggregates (Yu et al., 2017). It has been widely accepted that soil macroaggregates contain increasing pools of active and labile SOC that are derived dominantly from fresh SOC under manure treatments (Six et al., 2004; Jiang et al., 2018).

Microorganisms are considered as functionally diverse and fundamentally determine the development of soil aggregation as well as SOC accumulation and turnover. Definitely, the microstructural organization of soil macroaggregates provides a spatially heterogeneous environment for microorganisms, which are characterized by significant differences in the availability of numerous resources (e.g., SOC and nutrients), physical pore network, or predation pressures (Kravchenko et al., 2014; Rillig et al., 2017). Bacterial extracellular polysaccharides and fungal hyphae can attach to the microaggregates and bind them together to form the stable macroaggregates (Denef et al., 2001; De Gryze et al., 2005). Furthermore, arbuscular mycorrhizal fungi (AMF) contribute substantially to the formation of soil macroaggregates by SOC conservation (Wilson et al., 2009). To comprehensively understand SOC dynamics within soil macroaggregates, it is essential to determine the processes of stored SOC accumulation and turnover by measuring microbial carbon metabolism (Denef et al., 2009). The new and unstable SOC in the macroaggregates can facilitate microbial functions involved in carbon metabolism. Rapid turnover of SOC pool as a result of increased microbial carbon activity may lead to high nutrient availability for plants (Lehmann and Kleber, 2015). A growing body of evidence has demonstrated that the biomass and composition of microbial communities are viewed as the two key determinants of carbon metabolic activities at micrometer to millimeter scales (Schimel and Schaefer, 2012). Therefore, the variations in microbial biomass and community structure that occur in the interior and exterior surfaces of soil macroaggregates positively mediate carbon metabolism and consequently influence SOC sequestration and turnover (Nunan et al., 2003). To a large extent, a quantitative evaluation of how microbial carbon metabolism is influenced by microbial biomass and community structure will deepen our understanding of the main factors underpinning SOC turnover in soil macroaggregates.

As the most numerous and diverse group of soil microfauna, nematodes usually occupy multiple trophic levels in soil food webs and interact with microorganisms to affect carbon metabolic activity (Neher, 2010). The spatial distribution patterns of nematode assemblages in soil aggregates may be mainly controlled by the physical properties related to habitable pore systems (Baveye et al., 2018). The rapid development of micro-computed tomography provides the opportunity to nondestructively visualize and quantify the microhabitat of nematodes at micrometer resolutions (Peth et al., 2008). The relatively large pore sizes (>100 μm) of macroaggregates are beneficial for medium and large-sized nematodes passing through intra-aggregate pore spaces (Hassink et al., 1993). Nematodes usually have their own type of feeding apparatuses and food preferences, and the distinct feeding preferences of nematodes alter the compositions and functional characteristics of soil microbial communities (Griffiths, 1994; Liu et al., 2018). In general, microbial biomass and community structure are positively regulated by a strong top-down control of microfaunal predation (Neher, 2010). The highly complex network between nematodes and microorganisms in the macroaggregates has a pronounced influence on SOC turnover (Guan et al., 2018; Jiang et al., 2018). Nevertheless, a full understanding is still lacking to unravel the mechanisms of the linkages between nematodes and microorganisms driving SOC turnover in the macroaggregates.

Here, we focused on the biological interactions between nematodes and microorganisms governing SOC accumulation in soil macroaggregates. Specifically, this study was performed to test the following three hypotheses: (1) manure treatments increased SOC and nutrient availability of the macroaggregates and thereby stimulated the biomass of microbial communities; (2) manure treatments increased the abundance of large-size pores of the macroaggregates and subsequently enhanced the number of nematode assemblages; and (3) predation by nematodes on microbial communities improved microbial biomass and carbon metabolic activities. To this end, we conducted an 11-year field experiment under four manure application rates in a red soil. The macroaggregates with three replicates were separated from each treatment for physicochemical and biological analyses. The microstructures of soil macroaggregates were determined by micro-computed tomography and image analysis. The abundances and compositions of nematode assemblages and microbial communities were examined by microscopy and phospholipid fatty acid (PLFA) analysis, respectively. Microbial carbon metabolic capacity was determined by the Biolog EcoPlate system. Our results provide a deep insight into the important roles of the interactions between nematodes and microorganisms in determining SOC turnover in soil macroaggregates.

## Materials and methods

### Experimental site and design

The long-term fertilization experiment commenced at the Yingtan National Agroecosystem Field Experiment Station of the Chinese Academy of Sciences (28°15′20′′N, 116°55′30′′E) in Jiangxi Province, China. The experiment site has a typical subtropical climate with a mean annual temperature of 17.6°C and precipitation of 1,795 mm. The soil is derived from Quaternary red clay, which is classified as Ferric Acrisol according to the FAO classification system and Udic Ferralsol according to Chinese Soil Taxonomy. The long-term field experiment followed a completely randomized design with three replicates. The experiment was conducted since 2002, which consisted of 12 concrete plots with the following size: 2-m long, 2-m wide and 1.5-m deep. The four manure treatments were (1) no manure (M0); (2) low manure with 150 kg N ha^−1^ y^−1^ (M1); (3) high manure with 600 kg N ha^−1^ y^−1^ (M2); and (4) high manure with 600 kg N ha^−1^ y^−1^ and lime applied at 3,000 kg Ca (OH)_2_ ha^−1^ 3 y^−1^ (M3). Notably, the experiment was not a full factorial design as lime was only applied to the high manure treatment. Such an experimental design allowed us to determine the substantial effects of manure treatments on soil nematodes and microbial communities in the macroaggregates, while the impact of lime was just estimated under the high manure application. Pig manure had an average total carbon content of 386.5 g kg^−1^ and a total nitrogen content of 32.2 g kg^−1^ on a dry matter basis. The field was planted annually with corn monoculture (cultivar No. 11 from Denghai) from April to July. No management measures were taken with the exception of weeding by hand.

### Sampling and aggregate fractionation

The topsoil samples (0–20 cm) were collected after the harvest of maize in late July 2013. Ten soil cores were collected from each plot using an auger and pooled together to form a composite sample. Fresh samples were chilled on ice immediately after collection in the field and transported in a cooler to the laboratory. Soils were forced through a 4-mm sieve to remove visible residue and then homogenized. Soil was manually fractionated into three aggregate sizes, including large macroaggregates (>2 mm), small macroaggregates (0.25–2 mm), and microaggregates (<0.25 mm) (Jiang et al., 2013). In this study, only large macroaggregates were used for the subsequent stability analysis and micro-computed tomography scanning and thereafter referred to as macroaggregates. The macroaggregates were further subdivided into three subsamples for analyzing soil physicochemical properties, nematode assemblages, and microbial communities.

### Soil physical and chemical properties

Soil pH was determined using a glass electrode in a soil: water ratio of 1: 2.5 (w/v). Soil organic carbon and total nitrogen were determined by the Walkley-Black wet digestion method and the Kjeldahl method, respectively (Jackson, 1956; Nelson and Sommers, 1982). Cation exchange capacity was measured by the ammonium acetate method (Sumner and Miller, 1996). Dithionite-citrate-bicarbonate soluble Fe and oxalate-soluble Fe were detected using inductively coupled plasma optical emission spectrometry (PerkinEimer's, Optima 8000, USA). Water stability of soil macroaggregates was determined using a modification of the wet sieving method (Nimmo and Perkins, 2002) and was expressed as mean weight diameter. Tensile strength of 10 macroaggregates was detected by a crushing test using a uniaxial load frame (SANS CMT0104, Shenzhen, China) for evaluating mechanical stability (Dexter and Watts, 2001). The measurement and calculation of tensile strength were described in detail by Zhou et al. (2012).

### Micro-computed tomography scanning and image analysis

Macroaggregates (*n* = 5) were scanned using synchrotron-based micro-computed tomography at beam line BL13W1 at the Shanghai Synchrotron Radiation Facility. Detailed scanning settings and reconstruction procedures can be found in Zhou et al.'s (2013). The final 8-bit grayscale slices were stored in tiff format with a size of 1,024 × 1,024 × 1,024 voxels and a resolution of 3.7 μm. A cubic region of interest with a size of 600 × 600 × 600 voxels was selected from the central part of the soil macroaggregates for further analysis. Image preprocessing included removing the ring artifacts, enhancing the contrast, normalizing the brightness, and reducing noise with a 3D median filter (Zhou et al., 2013). The indicator kriging method was used to segment the voxels to pores and solids based on both the grayscale values and the spatial locations (Wang et al., 2011). The image-based porosity and pore size distribution of the segmented images were determined using the “opening” operation in the Quantim software (Vogel et al., 2010).

### Microbial phospholipid fatty acid (PLFA) analysis

To investigate soil microbial community, soil samples were characterized by PLFA analysis following a modified method described by Frostegård et al. (1993). Briefly, total lipids were extracted from 2 g of freeze-dried soil samples with a chloroform-methanol-citrate buffer solvent (1:2:0.8, v/v/v) and partitioned into neutral, glyco- and phospho-lipids by a silica acid column. Phospholipids were subjected to mild alkaline methanolysis and converted into fatty acid methyl esters prior to analysis. The fatty acid methyl esters were quantified based on the addition of the internal standard methyl nonadecanoate (19:0) by a HP 6890 Series gas chromatograph instrument equipped with a 7683 Series injector using helium as a carrier gas (Hewlett Packard, Wilmington, Delaware, USA).

Identification was performed using bacterial fatty acid standards and a software from the Microbial Identification System (MIS; Microbial ID Inc., Newark, DE, USA). Individual PLFAs were named using the standard nomenclature. Total microbial biomass was calculated by summing the mass of all the detected fatty acids (Bossio et al., 1998) and expressed as nmol PLFA g^−1^ dry soil. The sum of Gram-positive bacteria (GP, sum of all branched PLFAs) and Gram-negative bacteria (GN, sum of all monounsaturated PLFAs) was expressed as the total biomass (Frostegård and Bååth, 1996). From this value, the ratio of Gram-positive to Gram-negative bacteria (GP/GN) was calculated. The ratio of bacteria to fungi (B/F) was calculated by dividing the sum of bacterial PLFA markers by that of the fungal PLFA markers (18:1ω9 and 18:2ω6,9) (Zelles, 1997; Bååth and Anderson, 2003). The sum of PLFAs was further classified as actinomycetes (10Me PLFAs) (Frostegård et al., 1993) and AMF (16:1ω5) (Bach et al., 2010). Furthermore, two ratios of cyclopropyl PLFAs to their monoenoic precursors (cy17:0/16:1ω7 and cy19:0/18:1ω7) were used to indicate physiological or nutrient stress in the microbial communities (Kaur et al., 2005).

### Carbon metabolic profiles of microbial communities

The capability of soil microbial communities to utilize a variety of carbon sources was measured with Biolog EcoPlate (Biolog Inc., USA) (Zak et al., 1994). The Biolog EcoPlate system consisted of 31 different carbon sources plus a blank well in three replicates. The carbon sources were subdivided into six substrate groups including carbohydrates, carboxylic acids, amino acids, polymers, phenolic acids, and amines (Weber and Legge, 2009). Briefly, soil microorganisms were extracted as follows: 5 g soil (dry weight equivalent) was added to 45 ml sterile 0.85% (w/v) saline solution, and the mixture was shaken for 30 min at 90 rpm. Each well of the Biolog EcoPlate was inoculated with 200 μl of the mixed suspension and incubated at 25°C in the dark for 7 days. The average well color development (AWCD) reflecting carbon utilization was recorded as the optical density value at 590 nm with a plate reader at regular 24-h intervals. As previously described, the absorbance of a single time point at 96 h was used to compare the wells.

### Nematode assemblages

Nematodes were extracted from soil macroaggregates using a modified Baermann funnel method (Barker, 1985). Briefly, a handful (100 g) of soil was placed on a double cotton filter (Hygia rapid, Hartmann AG, Heidenheim, Germany) over a wire screen. Soil samples were placed on top of a Baermann funnel, which was filled with distilled water up to 1/4 of the core height. The nematodes were allowed to move through the filter into the distilled water in the funnel for 2 days at room temperature, and then clean suspensions were ready for the counting and identification of nematodes. Four functional groups of nematode assemblages, including bacterivores, fungivores, plant parasites, and omnivores and predators, were identified based on known feeding habits or stoma and esophageal morphology (Yeates et al., 1993). Nematode populations were counted and expressed as the number of nematodes per 100 g of dry weight soil.

### Statistical analyses

One-way analysis of variance (ANOVA) was used to test the effects of fertilization treatments on soil physicochemical properties, abundances of nematodes and microbial communities, and microbial carbon metabolism using Bonferroni's *post-hoc* test in the SPSS software (SPSS, Chicago, IL, USA). Principal coordinate analysis (PCoA) was performed to estimate the influence of fertilization on the Bray-Curtis distances of nematode and microbial community compositions (Anderson and Willis, 2003). We employed the “capscale” function for PCoA and “permutes” permutation-based testing for the calculation of the significance values.

Random forest modeling was conducted to quantitatively assess the important predictors of carbon metabolic capacity including soil properties, nematodes, and microbial community. The importance of each factor was evaluated by the increase in the mean square error between the observations and predictions (that is, the decrease in prediction accuracy) when the predictor was randomly permuted (Breiman, 2001). Random forest modeling was conducted using the randomForest package (Liaw and Wiener, 2002), and the significance of the model and predictor importance were determined using the A3R and rfPermute packages in the R software, respectively (Fortmann-Roe, 2013; Archer, 2016). Structural equation modeling was developed to uncover the direct and indirect contributions of soil physicochemical properties and nematodes to the microbial communities and carbon metabolic capacity. Structural equation modeling analysis was performed by the robust maximum likelihood estimation using AMOS 20.0 (Amos, Development Corporation, Meadville, PA, USA). The fitness of structural equation model was examined on the basis of nonsignificant chi-square test (*P* > 0.05), goodness of fit index, and the root mean square error of approximation (Hooper et al., 2008).

## Results

### Soil physicochemical properties and microbial carbon metabolism

**Figure 1:**
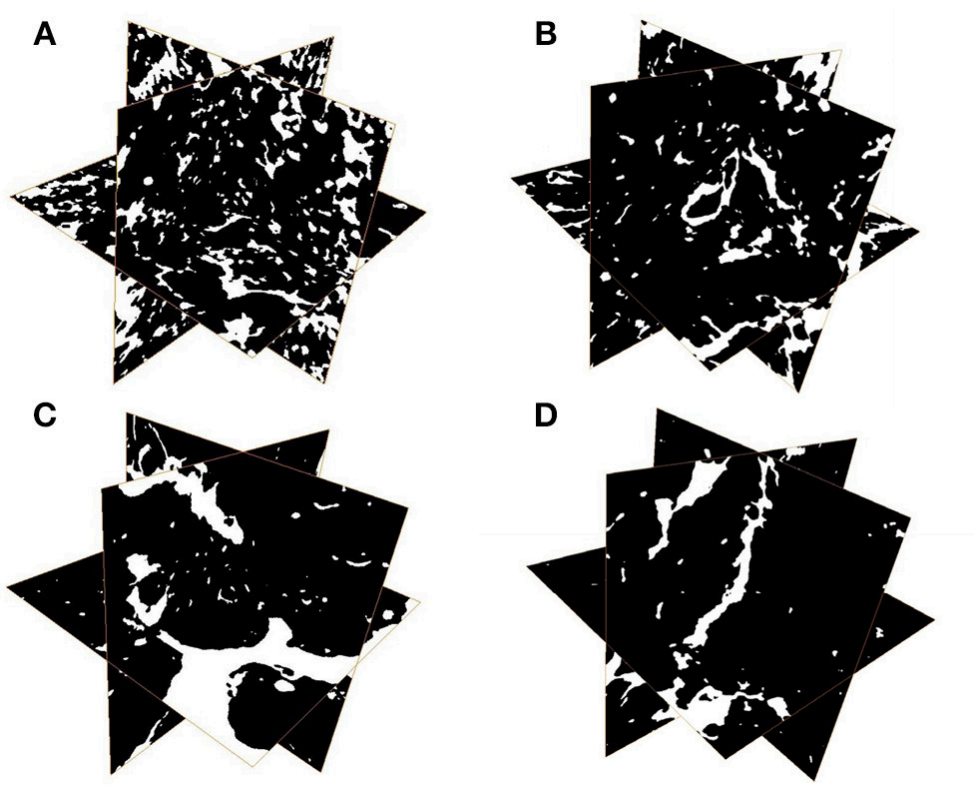
Representative 3D pore structures of soil macroaggregates under manure treatments. **(A)** No manure (M0), **(B)** low manure (M1), **(C)** high manure (M2), **(D)** high manure plus lime (M3).

**Figure 2:**
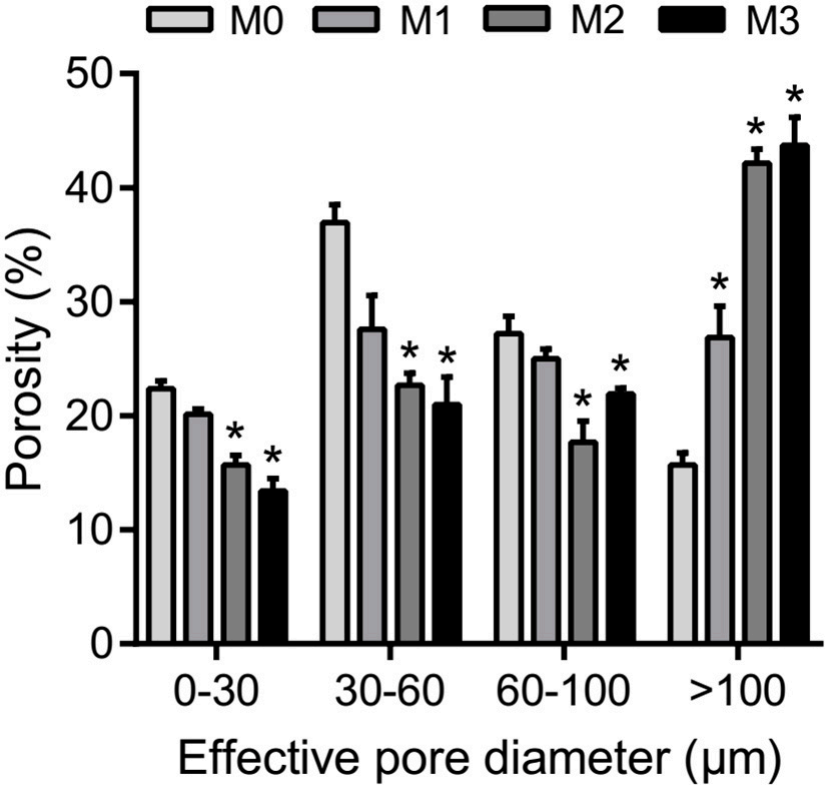
The pore size distribution in soil macroaggregates under manure treatments. Bars with * in the same pore diameter indicate significant differences (*P* < 0.05) between manure additions and the M0 treatment, as revealed by one-way ANOVA with Bonferroni's *post-hoc* test. M0, no manure; M1, low manure; M2, high manure; M3, high manure plus lime.

**Figure 3:**
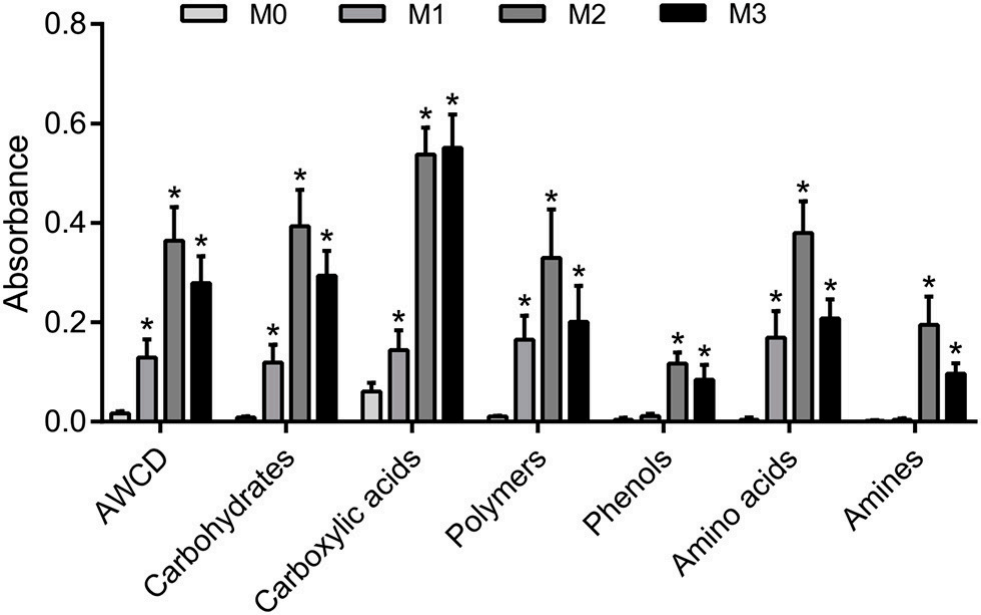
Soil carbon metabolism in soil macroaggregates under manure treatments. Microbial carbon metabolic activities in soil macroaggregates are reflected by the average well color development (AWCD) under manure treatments. The carbon sources are further subdivided into six substrate groups including carbohydrates, carboxylic acids, amino acids, polymers, phenolic acids, and amines. Bars with * indicate significant differences (*P* < 0.05) between manure additions and the M0 treatment, as revealed by one-way ANOVA with Bonferroni's *post-hoc* test. M0, no manure; M1, low manure; M2, high manure; M3, high manure plus lime.

The results of one-way ANOVA revealed that fertilization treatments significantly affected the physical and chemical properties of soil [*F*_(3, 8)_ = 7.49–73.87, *P* < 0.01]. The proportion of soil macroaggregate fraction was significantly increased by 30.6–33.7% and 37.8–40.6% under the high level manure treatments (M2 and M3) compared with that under low manure (M1) and no manure (M0) treatments. The M2 and M3 treatments were characterized by significantly (*P* < 0.05) higher pH, SOC, total nitrogen, cation exchange capacity, and oxalate-soluble Fe but lower dithionite-citrate-bicarbonate soluble Fe than the M0 and M1 treatments (Supplementary Table 1). Tensile strength and mean weight diameter were significantly (*P* < 0.05) elevated by high manure application. In addition, distinguishable differences in the 3D pore structure were found under the four fertilization treatments (Figure 1). The image-based intra-aggregate porosity substantially declined under the high manure treatments (Supplementary Table 1, *P* < 0.05). The pore size distribution was found to vary greatly under manure treatments. The proportion of the pore classes >100 μm in soil macroaggregates was significantly higher under the M2 (42.2%) and M3 (43.7%) treatments compared with that under the M0 (15.7%) and M1 (26.9%) treatments, while the proportions of the 0–30, 30–60, and 60–100 μm pore classes followed the opposite trend (Figure 2). Microbial carbon metabolism determined by the AWCD was significantly (*P* < 0.001) distinguished by fertilization regimes, following a general trend of M2 > M3 > M1 > M0 (Figure 3). The utilization of six substrate groups, including carbohydrates, carboxylic acids, amino acids, polymers, phenolic acids, and amines, followed a trend similar to that shown over the whole plate (Figure 3, *P* < 0.01).

### Microbial biomass and community structure

**Figure 4:**
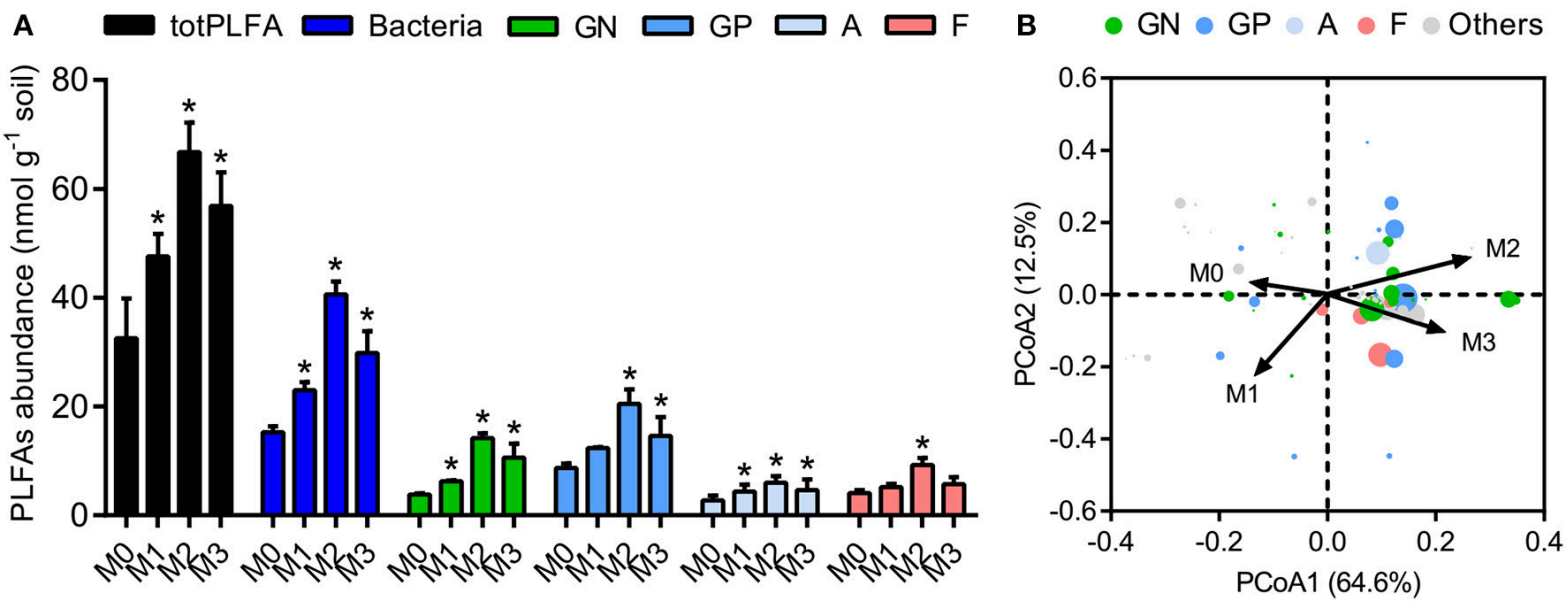
The biomasses and structures of microbial communities in soil macroaggregates under manure treatments. **(A)** The biomasses of total PLFA (totPLFA) and functional groups including bacteria, Gram-negative bacteria (GN), Gram-positive bacteria (GP), actinomycetes (A), and fungi (F). Bars with * indicate significant differences (*P* < 0.05) between manure additions and the M0 treatment, as revealed by one-way ANOVA with Bonferroni's *post-hoc* test. **(B)** The structure of microbial communities was significantly influenced by manure treatments, as indicated by Bray-Curtis distances using principal coordinate analysis (PCoA). M0, no manure; M1, low manure; M2, high manure; M3, high manure plus lime.

We determined the microbial biomass and community structure in soil macroaggregates by PLFA analysis. The total biomass varied considerably in response to manure treatments and was significantly higher under manure treatments (M1, M2, and M3) than that under the M0 treatment (Figure 4, *P* < 0.05). The general pattern of M2 > M3 > M1 > M0 was observed in specific PLFA signatures, such as bacteria, fungi, Gram-negative bacteria, Gram-positive bacteria, and actinomycetes (Figure 4). The distribution of microbial PLFA biomarkers as an index of community structure exhibited a substantial response to manure treatments. Principal coordinate analysis revealed that the microbial community structure under the high level of manure addition was distinguished clearly from that under the M0 and M1 treatments (Figure 4, *P* < 0.001). The ratio of B/F increased significantly, but the ratio of GP/GN decreased with the increase in manure addition (Supplementary Figure 1, *P* < 0.05). The ratios of cyclopropyl fatty acids to their precursors (17:0 cyclopropyl per precursor and 19:0 cyclopropyl per precursor) calculated as two stress indexes were in the microbial communities. The ratios of 17:0 cyclopropyl to 16:1ω7 (cy17:0/16:1ω7c) and 19:0 cyclopropyl to 18:1ω7 (cy19:0/18:1ω7c) under the high manure treatments were approximately 4.0–5.9-fold lower than those under the M0 treatment and 1.6–2.1-fold lower than those under the M1 treatment (Supplementary Figure 1).

### Nematode assemblages

**Figure 5:**
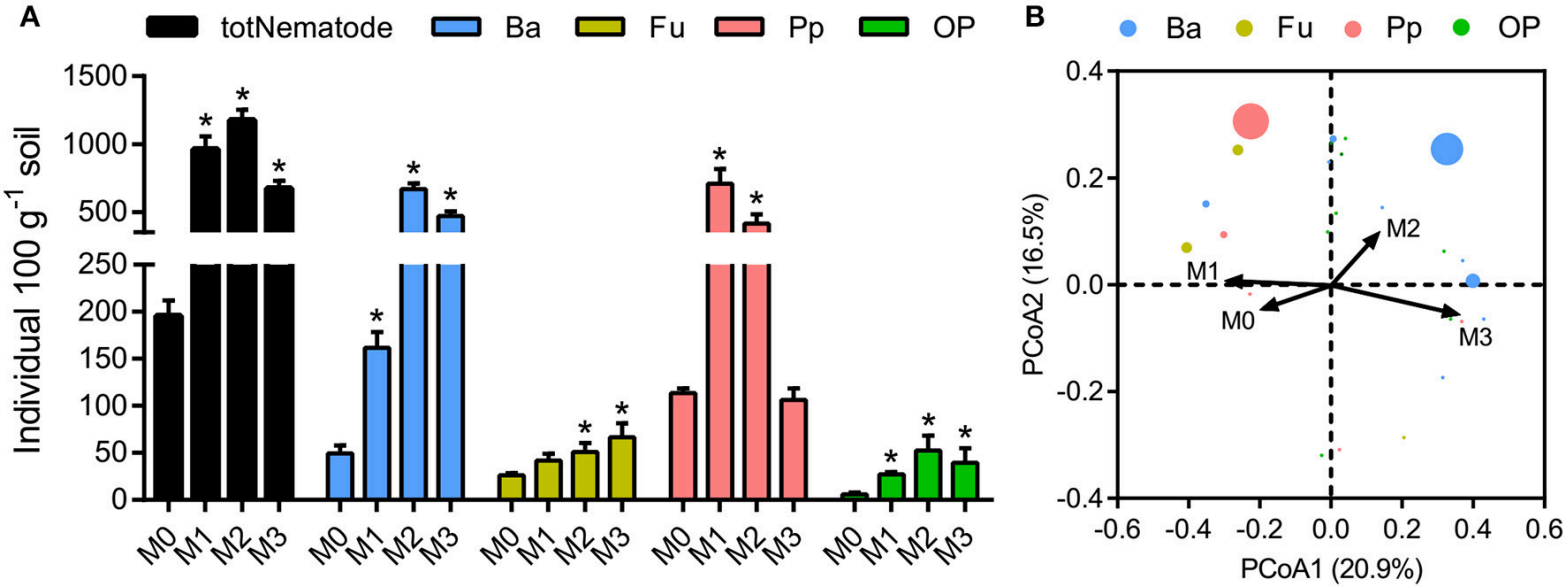
The numbers and structures of nematode assemblages in soil macroaggregates under manure treatments. **(A)** The numbers of total nematodes (totNematode) and functional groups including bacterivores (Ba), fungivores (Fu), plant parasites (Pp), and omnivores and predators (OP). Bars with * indicate significant differences (*P* < 0.05) between manure additions and the M0 treatment, as revealed by one-way ANOVA with Bonferroni's *post-hoc* test. **(B)** The structures of nematode assemblages were well separated by manure treatments based on Bray-Curtis distances using principal coordinate analysis (PCoA). M0, no manure; M1, low manure; M2, high manure; M3, high manure plus lime.

The total number of nematodes in soil macroaggregates was counted under fertilization treatments, as well as four functional groups including bacterivores, plant parasites, fungivores, as well as omnivores and predators. Manure application considerably (*P* < 0.01) affected the number and composition of nematode assemblages. Bacterivores (46.6%) and plant parasites (30.5%) were the two most abundant trophic groups in the nematode assemblages (Supplementary Figure 2). Manure treatments significantly increased the total number of nematodes and the four functional groups in soil macroaggregates (Figure 5, *P* < 0.05). However, the elevated soil pH by lime reduced the total number of nematodes as well as bacterivores and plant parasites (Figure 5). Principal coordinate analysis revealed that the nematode assemblages were well segregated by fertilization treatments in regard to the abundances of bacterivores and plant parasites (Figure 5, *P* < 0.001). Plant parasites were enriched under the M1 treatment, comprising 73.5% of the total number, while bacterivores were dominated under the M2 and M3 treatments, comprising 57.1 and 69.9% of the total number, respectively (Supplementary Figure 2).

### Microbial carbon metabolism affected by soil properties, nematodes, and microbial communities

Bacterial and fungal biomass was positively associated with soil pH (*r* = 0.885, *P* < 0.001 and *r* = 0.665, *P* = 0.018) and SOC (*r* = 0.876, *P* < 0.001 and *r* = 0.598, *P* = 0.040), as well as microbial carbon metabolic function (*r* = 0.892, *P* < 0.001 and *r* = 0.771, *P* = 0.003). The proportion of pores with size >100 μm was significantly associated with bacterivores (*r* = 0.915, *P* < 0.001) and fungivores (*r* = 0.841, *P* = 0.001), as well as omnivores and predators (*r* = 0.881, *P* < 0.001). The abundance of AMF was significantly related to mean weight diameter (*r* = 0.641, *P* = 0.025). The abundance of bacterivores was closely correlated with bacterial biomass (*r* = 0.917, *P* < 0.001), Gram-negative bacteria (*r* = 0.960, *P* < 0.001), and microbial carbon metabolism (*r* = 0.947, *P* < 0.001). However, the abundance of bacterivores exhibited a marginal relationship with actinomycetes (*r* = 0.572, *P* = 0.052). The abundance of fungivores was highly correlated with AMF abundance (AMF, *r* = 0.693, *P* = 0.012) but showed no significant relationship with fungal biomass (*r* = 0.558, *P* = 0.059).

**Figure 6:**
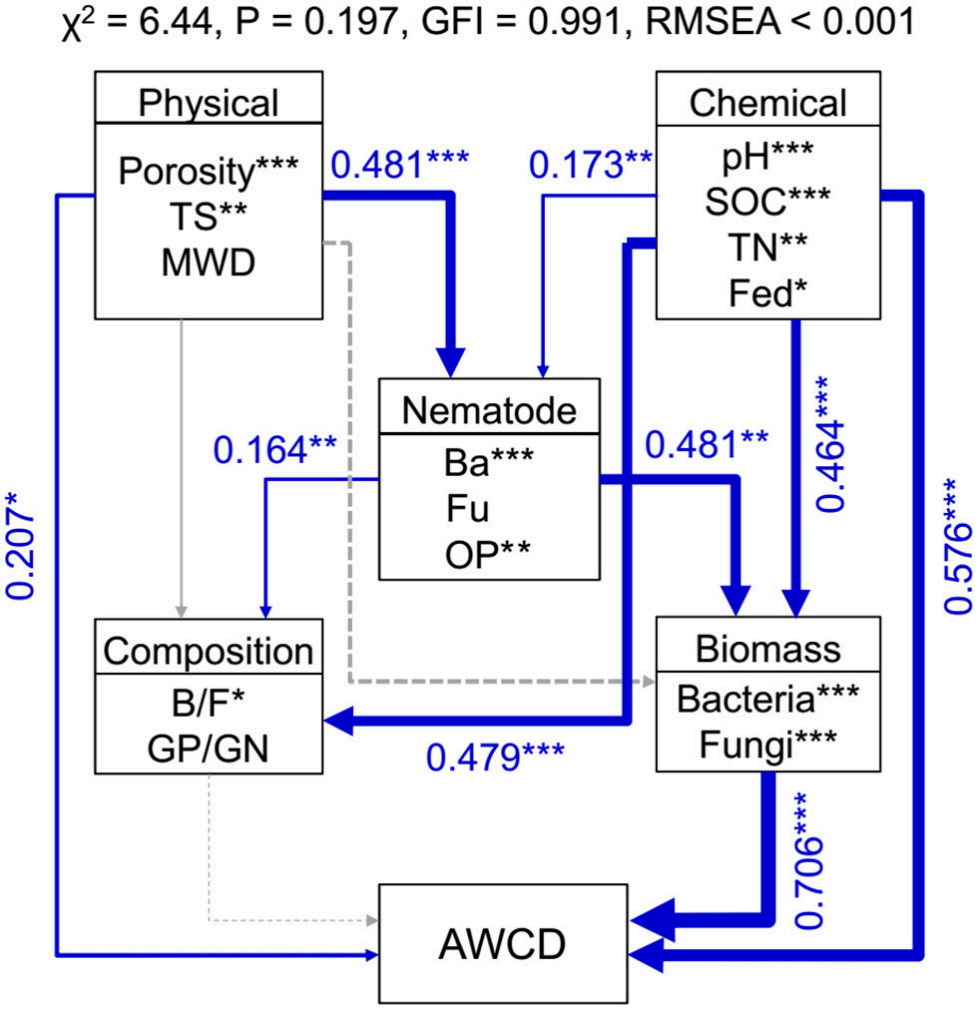
Structural equation modeling shows the direct and indirect effects of soil properties and nematodes on bacterial biomass, community structure, and carbon metabolic activities in soil macroaggregates. Microbial carbon metabolism was reflected by the average well color development (AWCD). Blue lines indicate positive effects. The width of arrows indicates the strength of significant standardized path coefficients. Paths with non-significant coefficients are presented as gray lines. The fitness of structural equation modeling is assessed on the basis of non-significant χ-square test (*P* > 0.05), goodness-of-fit index (GFI), and the root mean square error of approximation (RMSEA). SOC, soil organic carbon; TN, total nitrogen; Fed, dithionite-citrate-bicarbonate soluble Fe; TS, Tensile strength; MWD, mean weight diameter; Ba, bacterivores; Fu, fungivores; OP, omnivores and predators; B/F, the ratio of bacteria to fungi; GP/GN, the ratio of Gram-positive bacteria to Gram-negative bacteria. ***P < 0.001, **P < 0.01, *P < 0.05.

Random forest modeling was performed to identify the important predictors of nematode assemblages, microbial communities, and carbon metabolic activities. The models were significant at the 0.01 level with *R*^2^ = 0.56–0.92. Random forest modeling indicated that soil pH and SOC were the two greatest determinants of microbial communities (12.8–27.2% and 11.7–15.5%) and carbon metabolism (15.7 and 10.8%) (Table 1). However, total porosity (13.0–21.3%) exhibited a stronger effect on nematode assemblages than SOC (8.7–19.1%) and pH (6.8–19.8%), followed by tensile strength (7.1–15.2%) and mean weight diameter (1.6–9.6%) (Table 1). Similarly, the predation of bacterivores suggested a dramatic impact on microbial carbon metabolism (6.2–13.8% and 8.4%) (Table 1). Structural equation modeling was used to assess the direct and indirect effects of soil physicochemical properties and nematode predation on the microbial community structure and carbon metabolic profiles. We found that the effect of soil chemical variables on carbon metabolic activities was higher than that of physical features (Figure 6). Microbial carbon metabolism was significantly related to microbial biomass rather than community structure. However, soil physical properties showed a primary effect on nematode assemblages, while chemical characteristics made a pronounced contribution to microbial biomass and community structure (Figure 6). Notably, nematode predation may exhibit an indirect positive influence on microbial carbon metabolism by promoting microbial biomass.

**Table 1:** Relationships between soil properties, nematodes, microbial community and AWCD based on random forest analysis in the macroaggregates.

	**Nematode assemblage**					**Microbial community**					**AWCD**
	**totNematode**	**Ba**	**Fu**	**Pp**	**OP**	**totPFLA**	**Bacteria**	**Fungi**	**B/F**	**GP/GN**
**SOIL PROPERTIES**											
pH	13.25^**^	8.18^*^	6.80^*^	19.84^**^	8.68^*^	16.20^**^	27.19^**^	17.55^**^	12.82^**^	15.13^**^	15.66^**^
SOC	19.08^**^	16.66^**^	8.69^*^	17.32^**^	12.48^**^	13.07^**^	11.72^**^	15.47^**^	12.05^**^	12.46^**^	10.79^**^
TN	10.98^**^	9.92^**^	4.27	11.48^**^	9.70^*^	8.82^*^	7.67^*^	4.95	6.42^*^	10.79^**^	5.20^*^
CEC	1.00	3.57	6.25	6.46	2.20	0.97	4.27	3.48	2.77	2.63	4.97
Feo	6.58	5.83	4.64	3.28	1.98	8.34^*^	4.39	1.78	6.01	3.23	0.61
Fed	4.47	10.99^*^	0.97	5.21	3.24	10.21^**^	6.25^*^	4.30	10.45^**^	2.06	7.87^*^
Porosity	21.27^**^	20.56^**^	18.09^**^	17.25^**^	13.01^**^	6.95^*^	9.97^*^	2.37	6.06^*^	6.75	7.61^*^
Tensile strength	9.82^*^	15.19^**^	7.09^*^	9.86^*^	10.31^**^	5.07	5.77	8.75^*^	3.55	12.05^**^	5.72^*^
MWD	2.79	3.98	9.57^*^	2.40	1.62	5.74	5.44	2.47	0.36	0.89	0.93
**NEMATODE ASSEMBLAGE**											
Ba	–	–	–	–	–	9.38^**^	13.83^**^	6.20	7.04^*^	10.96^**^	8.42^*^
Fu	–	–	–	–	–	5.77	4.67	1.64	0.34	2.63	1.28
Pp	–	–	–	–	–	2.06	3.11	7.50^*^	1.05	0.33	4.40
OP	–	–	–	–	–	7.61^*^	11.24^**^	7.28^*^	5.06^*^	5.38	4.75^*^
**MICROBIAL COMMUNITY**											
Bacteria	–	–	–	–	–	–	–	–	–	–	10.33^**^
Fungi	–	–	–	–	–	–	–	–	–	–	6.04^*^
B/F	–	–	–	–	–	–	–	–	–	–	6.48^*^
GP/GN	–	–	–	–	–	–	–	–	–	–	2.66

*Microbial metabolic activities are reflected by the average well color development (AWCD). Soil physicochemical properties are indicated by pH, soil organic carbon (SOC), total nitrogen (TN), cation exchange capacity (CEC), oxalate soluble Fe (Feo), dithionite-citrate-bicarbonate soluble Fe (Fed), porosity, tensile strength, and mean weight diameter (MWD). Nematode assemblage is indicated by total number of nematodes (totNematode), bacterivores (Ba), fungivores (Fu), plant parasites (Pp), as well as omnivores and predators (OP). Microbial community is indicated by total PLFA (totPLFA), bacteria, fungi, and the ratios of bacteria to fungi (B/F) and Gram-positive bacteria to Gram-negative bacteria (GP/GN). Bold values denote the significant relationships between soil properties, nematodes, microbial community and AWCD. –, not calculated*;

** *P < 0.01*;

* *P < 0.05*.

## Discussion

### Microbial communities in the macroaggregates under fertilization treatments

The biomarker PLFA analysis highlighted the significant differences in microbial biomass and community composition under different fertilization treatments. We observed that the spatial distribution of soil microbial biomass in macroaggregates clearly reflected the uniform pattern. The biomasses of total microorganisms and microbial functional groups were simultaneously enriched by the higher SOC and pH because of fresh organic matter inputs (Figure 4). Fertilization treatments play a key role in diversifying the microhabitats of soil macroaggregates; the chemical conditions (e.g., pH, SOC, and total nitrogen) of which represent an important aspect of niche differentiation that selects different functional groups (Briar et al., 2011; Jiang et al., 2018). Moreover, amorphous minerals have high surface areas to adsorb SOC, which affects the turnover rates of SOC pools and the availability of energy to soil microorganisms (Torn et al., 1997). The results suggested that these groups were sensitive indicators of the substantial increases in soil pH, SOC, and nutrient availability under fertilization treatments.

Manure treatments drastically altered the microbial community composition in terms of the ratios of B/F and GP/GN. The observed changes in soil microbial community structure revealed that the abundance of functional groups was unevenly distributed under management regimes (Supplementary Figure 1). More specifically, the increasing B/F ratio was largely governed by the faster increase in bacterial abundance than fungal abundance. The high bacterial abundance contributes prominently to SOC turnover and the liberation of nutrients and improves the overall quality (Smith et al., 2014). However, fungi play a crucial role in both the physical and chemical fertility of soil through aggregation and mycorrhizal associations, respectively. For instance, the fungal hyphal length is greater in aggregated soil than in sieved soil, emphasizing the interdependence between soil structure and microorganisms. Fungal hyphae have been shown to enmesh microaggregates into larger macroaggregates and facilitate the formation of macroaggregates (De Gryze et al., 2005). The extensive mycelial network of AMF produces large quantities of a sticky proteinaceous hydrophobic substance (glomalin-related soil protein) and recalcitrant polymers (chitin and chitosan) (Rillig and Mummey, 2006). Our results suggested that the increase in AMF abundance could improve the mean weight diameter, which was an integrated measure of soil aggregate structure and stability. The increase in AMF abundance suggests that the symbiosis of AMF with plants is gradually established and concomitantly beneficial for plant growth (Montesinos-Navarro et al., 2012). Furthermore, the decrease in the GP/GN ratio suggests that Gram-negative bacteria play a relatively important role in manured soils. Proteobacteria, including Alphaproteobacteria, Betaproteobacteria, and Gammaproteobacteria, are enriched in the macroaggregates that typically contain alkyl and aliphatic groups (Janik et al., 2007). It is well established that closely interconnected Gram-negative bacteria (e.g., *Burkholderia, Rhizobiales, Rhodospirillales*, and *Pseudomonas*) frequently occupy a wide range of ecological niches and exhibit cooperative partnerships together with plants (Coenye and Vandamme, 2003; Jiang et al., 2017). The complex coupling patterns of these specific genera reflect synergistic relationships to alleviate abiotic stresses and promote plant growth in many crop systems (Bouizgarne et al., 2015). Notably, manure treatments decreased the ratios of cy17:0/16:1ω7c and cy19:0/18:1ω7c, suggesting that pH and nutritional stress were less limiting for the growth of the microbial population. Two ratios of cy per precursor have been proposed as indicators of starvation and pH stress to the microbial community (Moore-Kucera and Dick, 2008). Lower values of these ratios are associated with increased SOC supplementation and rapid rates of microbial growth and multiplication (Sampedro et al., 2009).

### Nematode assemblages in the macroaggregates under fertilization treatments

Manure treatments enhanced the total amount of nematodes in soil macroaggregates compared to the M0 treatment, which was principally caused by explosive increases in bacterivores under the M2 and M3 treatments (Figure 5). We observed that soil porosity exhibited a major effect on the structures of soil nematode assemblages (Figure 6, Table 1). Changes in soil nematode assemblages corresponded largely to those in soil porosity of the macroaggregates. The porosity of soil macroaggregates with porosity >100 μm was significantly improved under the M2 and M3 treatments (Figure 2), likely supporting the observed high densities of bacterivores. Nematodes are typically sensitive to the changes in the proportions of large pore sizes that are associated with intensive management (Cardoso et al., 2012). The intra-aggregate microarchitecture of soil macroaggregates with an adequate pore size range offers a suitable space for nematodes moving and migrating to their food sources. The percentage of soil macoraggregates with pore size >100 μm was an important determinant of the activity and survival of nematodes with large body sizes. Nematodes are aquatic organisms that depend primarily on thin water films to live and move through existing pathways of habitable soil pores with diameters of 30–90 μm (Hassink et al., 1993). However, the pores with diameters of 200–1,000 μm are much larger than the diameters of nematodes, which may constrain colonization and habitation by nematodes in soil macroaggregates (Quénéhervé and Chotte, 1996). Furthermore, the abundance of bacterivorous nematodes theoretically provides information about bacterial contributions to the decomposition and turnover of soil organic matter (Vestergård, 2004). The high amounts of bacterial-feeding nematodes under high manure treatments implied that the bacterial-based energy channel contributed strongly to SOC decomposition. In marked contrast, the increased pH with lime seemed to inhibit the total nematode density, which was particularly caused by a sharp decline in plant parasites under the M3 treatment (Figure 5). Ammonia has been reported as a nematicide to effectively suppress plant-parasitic nematodes with the application of organic manure and lime (Ben-Yephet et al., 2006). Acid-base neutralization at neutral pH values can trigger a shift in the equilibrium from ammonium toward ammonia and enhance the nematicidal activities of controlling nematodes (Oka et al., 2007). Despite the moderate influence of pH, the abundance of plant parasites would plummet in response to neutral conditions as a result of the increased nematicidal activity (Oka et al., 2006; Jiang et al., 2013).

### Nematode-microorganism interactions stimulate microbial carbon metabolism

Our study took a further step toward describing the intimate interactions between nematodes and microorganisms in macroaggregates. The high value of the nematode structure index in macroaggregates suggests a complex community structure with many linkages in soil food web (Jiang et al., 2013). The positive bacterivore-bacteria relationship suggested that predation by bacterivores stimulates the increase in bacterial biomass. Recently, the incubation experiments have reported that the effects of nematode grazers on microbial biomass range from positive to negative (Trap et al., 2016). The slight to moderate grazing of bacterivores exerts positive feedback on bacterial biomass and activity (Fu et al., 2005). However, the population developments of each nematode species differed according to the prey bacteria. This study suggested that bacterivores showed strongly positive relationships with total bacteria and Gram-negative bacteria rather than actinomycetes. Bacterivores do not multiply in the presence of the filamentous bacteria *Actinomyces* sp. and the Gram-positive bacteria *Arthrobacter* sp. (Buchan et al., 2013). Large and filamentous bacterial cells can escape uptake by nematodes as a result of their small buccal cavity (Venette and Ferris, 1998). Gram-positive bacteria are likely to be less suitable food for protozoans and nematodes than Gram-negative bacteria. The lower edibility of Gram-positive bacteria may be related to a lower rate of digestion of their cell wall, which enables survival during passage through microbial grazers (Rønn et al., 2012).

Nematodes hold a central position in soil food webs, and the population dynamics of nematode communities are closely linked to SOC dynamics. The presence of bacterivorous nematodes substantially promotes microbial basal respiration contributing to SOC dynamics (Neher, 2010). Structural equation modeling quantitatively described that bacterivores may exert a stronger impact on microbial biomass than community structure and eventually increase microbial carbon metabolism in the macroaggregates (Figure 6). Our previous result reported that the predation of bacterivores exhibited indirectly positive associations with the sizes and turnover rates of SOC pools in the macroaggregates (Jiang et al., 2018). Microorganisms can dramatically increase SOC retention by directly incorporating living and senescing fungal and bacterial biomass into the stable SOC reservoir (Liang et al., 2017). Moreover, selective feeding by bacterivores on active bacteria suppresses soil metabolic quotient and thus stimulates SOC accumulation in the macroaggregates (Jiang et al., 2013). Therefore, this study supported that increased bacterivore abundance enhanced the grazing pressure on microorganisms and concurrently promoted microbial-derived SOC turnover. In addition, AMF generally prefer to grow in the macroaggregates with high porosity to facilitate SOC stabilization and protection by enhancing well-developed soil aggregation. The contribution of AMF hyphae to SOC sequestration contains extra-radical hyphae as well as glomalin-related soil proteins produced by the hyphae (González-Chávez et al., 2010). Glomalin is considered an important pool of recalcitrant SOC with a relatively long turnover time (Driver et al., 2005). Furthermore, AMF that form mutualistic associations with plants are vulnerable to grazing by fungivorous nematodes. Fungivores *Aphelenchoides* sp. populations grow rapidly on saprophytic (*Agrocybe* and *Chaetomium*) and mycorrhizal (*Cenococcum, Hymenoscyphus*, and *Laccaria*) fungi (Ruess et al., 2000). Furthermore, the predation by fungivorous nematodes on AMF *Glomus* spp. and *Acaulospora* spp. shows a positive feedback effect on AMF abundance (Hua et al., 2010). Collectively, soil macroaggregates could be identified as a well-organized soil food web with interrelated functional relationships between nematodes and microorganisms in regulating SOC turnover.

## Conclusions

In summary, we observed that fertilization regimes considerably affected nematode assemblages, microbial communities, and carbon metabolic activities in soil macroaggregates. The abundances of nematode and microbial communities were significantly increased under manure treatments. Soil physical properties (soil porosity) showed an overwhelming effect on nematode assemblages, while chemical characteristics (SOC and pH) made a pronounced contribution to the microbial community and carbon metabolic capacity. Moreover, bacterivores may exert an indirect positive effect on carbon metabolism predominantly via changes in the microbial biomass. Our study highlighted the important role of the biological linkages between nematodes and microorganisms in mediating SOC turnover in soil macroaggregates. In the future, an explicit representation of the nematode-microorganism interactions affecting SOC dynamics will be required to promote new interdisciplinary research and stimulate novel theoretical developments.

## Author contributions

YJ, HZ, and BS designed all the experiments and wrote the manuscript. YJ, HZ, LC, and HF were responsible for performing the field and lab experiments. All authors analyzed all data and discussed the results.

### Conflict of interest statement

The authors declare that the research was conducted in the absence of any commercial or financial relationships that could be construed as a potential conflict of interest.
