# Supplementary material for: Nematodes and Microorganisms Interactively Stimulate Soil Organic Carbon Turnover in the Macroaggregates
Source: Front Microbiol. 2018 Nov 26;9:2803. doi: 10.3389/fmicb.2018.02803 (PMC6275192; doi:10.3389/fmicb.2018.02803)
Supplement: Supplementary file 1 [file Table_1.docx]

**Supplementary Figures and Tables**

**Nematodes and Microorganisms Interactively Stimulate Soil Organic Carbon Turnover in the Macroaggregates**

***Yuji Jiang^1,^ ^†^, Hu Zhou^1, †^, Lijun Chen^1,2^, Ye Yuan^1,3^, Huan Fang^1,2^, Lu Luan^1,2^, Yan Chen^1^, Xiaoyue Wang^1^, Manqiang Liu^4^, Huixin Li^4^, Xinhua Peng^1^, Bo Sun^1,^****

*^1^ State Key Laboratory of Soil and Sustainable Agriculture, Institute of Soil Science, Chinese Academy of Sciences, Nanjing, China, ^2^ University of Chinese Academy of Sciences, Beijing, China, ^3^ Mudanjang Tobacco Science Research Institute, Harbin, China, ^4^ College of Resources and Environmental Sciences, Nanjing Agricultural University, Nanjing, China.*


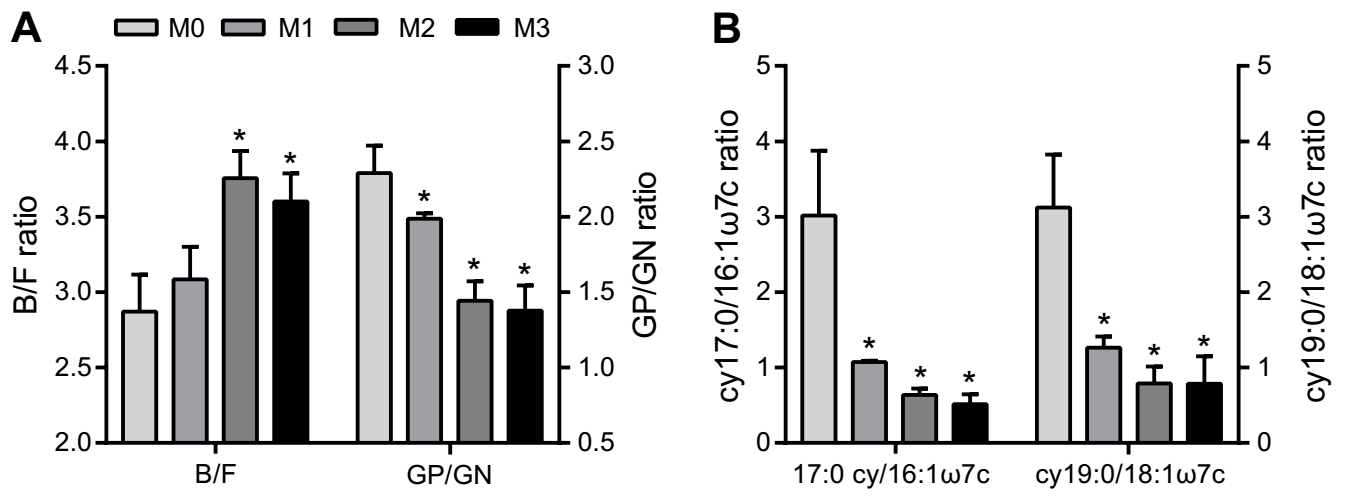


**FIGURE S1│The composition and two stress indexes of microbial communities in soil macroaggregates under manure treatments.** **(A)** The composition of microbial communities is indicated by the ratios of bacteria to fungi (B/F) and Gram-positive bacteria to Gram-negative bacteria (GP/GN). **(B)** Microbial stress indexes are indicated by the ratios of 17:0 cy to 16:1ω7 (cy17:0/16:1ω7c) and 19:0 cy to 18:1ω7 (cy19:0/18:1ω7c). Bars with ***** indicate significant differences (*P* < 0.05) across soil profiles, as revealed by one-way ANOVA with Bonferroni’s post hoc test. M0, no manure; M1, low manure; M2, high manure; M3, high manure plus lime.


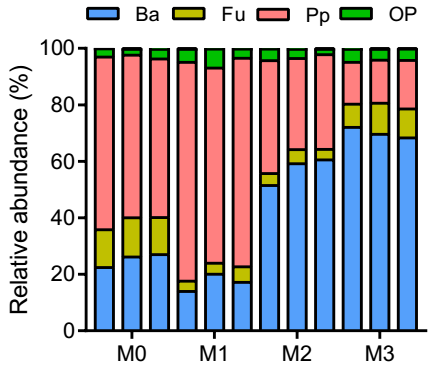


**FIGURE S2│The composition of nematode assemblages in soil macroaggregates under manure treatments.** Ba, bacterivores; Fu, fungivores; Pp, plant parasites; OP, omnivores and predators. M0, no manure; M1, low manure; M2, high manure; M3, high manure plus lime.

**TABLE S1│**Soil physicochemical properties of soil macroaggregates under four fertilization treatments.

| **Physicochemical properties** | **M0** | **M1** | **M2** | **M3** |
| --- | --- | --- | --- | --- |
| Fraction proportion (%) | 40.5 ± 2.7^a^ | 45.2 ± 3.6^a^ | 65.1 ± 3.3^b^ | 68.2 ± 5.4^b^ |
| pH | 4.64 ± 0.01^a^ | 4.88 ± 0.03^b^ | 5.77 ± 0.05^c^ | 6.68 ± 0.06^d^ |
| SOC (g/kg) | 3.67 ± 0.13^a^ | 6.93 ± 0.31^b^ | 9.38 ± 0.35^c^ | 8.95 ± 0.36^c^ |
| TN (g/kg) | 0.49 ± 0.02^a^ | 0.76 ± 0.02^b^ | 0.98 ± 0.03^c^ | 0.96 ± 0.03^c^ |
| CEC (cmol kg^–1^) | 14.54 ± 0.34^a^ | 14.98 ± 0.12^a^ | 16.94 ± 0.22^b^ | 16.50 ± 0.55^b^ |
| Feo (g kg^–1^) | 1.35 ± 0.03^a^ | 1.54 ± 0.05^b^ | 1.72 ± 0.02b^c^ | 1.68 ± 0.06^c^ |
| Fed (g kg^–1^) | 54.68 ± 0.47^b^ | 53.35 ± 0.59^b^ | 50.85 ± 0.34^a^ | 49.26 ± 0.26^a^ |
| TS (KPa) | 350 ± 32^a^ | 395 ± 47^a^ | 708 ± 23^b^ | 869 ± 58^c^ |
| MWD (mm) | 0.85 ± 0.04^a^ | 1.12 ± 0.11^ab^ | 1.41 ± 0.02^bc^ | 1.65 ± 0.22^c^ |
| Porosity | 0.18 ± 0.10^c^ | 0.12 ± 0.01^b^ | 0.07 ± 0.01^a^ | 0.10 ± 0.01^ab^ |

*Values are the means (n =3) ± the standard error. Values in the same row within soil macroaggregates followed by a different lowercase letter indicate a significant (P < 0.05) difference by Bonferroni's post-hoc test. SOC, soil organic carbon; TN, total nitrogen; CEC, cation exchange capacity; Feo, oxalate soluble Fe; Fed, dithionite-citrate-bicarbonate soluble Fe; TS, Tensile strength; MWD, mean weight diameter.* *M0, no manure; M1, low manure; M2, high manure; M3, high manure plus lime.*
